# Two Decades of Same-Sex Marriage in Sweden: A Demographic Account of Developments in Marriage, Childbearing, and Divorce

**DOI:** 10.1007/s13524-019-00847-6

**Published:** 2020-01-09

**Authors:** Martin Kolk, Gunnar Andersson

**Affiliations:** 1 Demography Unit, Department of Sociology, Stockholm University, Stockholm, Sweden; 2 Stockholm University Center for Cultural Evolution, Stockholm University, Stockholm, Sweden; 3 Institute for Future Studies, Stockholm, Sweden

**Keywords:** Same-sex marriage, Registered partnership, Divorce, Childbearing, Sweden

## Abstract

In this study, we provide demographic insight into the still relatively new family form of same-sex marriage. We focus on period trends in same-sex marriage formation and divorce during 1995–2012 in Sweden and the role of childbearing in same-sex unions. The period begins with the introduction of registered partnership for same-sex couples and also covers the introduction of formal same-sex marriage in 2009. We use register data for the complete population of Sweden to contrast patterns in male and female same-sex marriage formation and divorce. We show that female same-sex union formation increased rapidly over the period, while trends for male same-sex unions increased less. The introduction of same-sex marriage legislation in 2009 appears to have had little effect on the pace of formation of same-sex unions. In contrast, legal changes supporting parental rights in same-sex unions may have fueled the formation of female same-sex marriages as well as parenthood in such unions. Further, we show that divorce risks in the marital unions of two women are much higher than in other types of marriages. We find some convergence of divorce risks across union types at the end of our study period: male same-sex unions have the same divorce risk levels as opposite-sex marriages, and the elevated risks of divorce in female same-sex unions appear to have stabilized at somewhat lower levels than those observed in the late 1990s.

**Electronic supplementary material:**

The online version of this chapter (10.1007/s13524-019-00847-6) contains supplementary material, which is available to authorized users.

## Introduction

Sweden is often considered a forerunner in family change regarding many aspects of the so-called second demographic transition of increased diversity in family dynamics. New family trends have often appeared in Sweden and the other Nordic countries, to later be observed across Europe and other developed countries (van de Kaa 1987:11). Whether this is a general pattern may be debatable, but it certainly holds for the development of same-sex unions and the legal recognition of same-sex marriages. This family form first gained legal recognition in the Nordic countries and shortly thereafter became established in a wider range of countries across Europe and beyond.

This family form is still relatively new, but it has existed in Sweden for more than two decades. Data on same-sex partnerships and marriages in Sweden are available through the country’s extensive population-register system. This makes it possible to carry out a demographic survey of the patterns in same-sex marriage formation and marriage dissolution for a period that covers more than just the initial phase of very new same-sex marriages. Our study expands on previous demographic research on same-sex marriages in Scandinavia by Andersson et al. (2006), Andersson and Noack (2010), and Wiik et al. (2014), with the goal of determining whether previously observed patterns of same-sex marriage dynamics reflected any long-standing patterns in marriage formation and divorce or rather those dominated by the pioneers of same-sex spouses. We further examine the increasingly important role of childbearing in same-sex union dynamics. Earlier findings indicated a crossover from a dominance of male couples to that of female unions in same-sex marriage formation; they also demonstrated elevated divorce risks in same-sex marriages of women (Andersson et al. 2006; Wiik et al. 2014). In our study, we aim to provide better insight into the situation in Sweden based on data covering a longer period and focusing on changes in patterns over time.

Our study is based on high-quality longitudinal data spanning 1995–2012 that are analyzed through the application of appropriate demographic methods. The field of same-sex family dynamics is a rapidly growing field of research but is often based on relatively sketchy quantitative data. In particular, there is still a dearth of reliable quantitative data on and demographic analyses of the family dynamics in relation to childbearing and parenthood in same-sex unions. A growing body of research has focused on children living in same-sex union households, but hardly any research has attended to period trends in childbearing within those unions.

In terms of social context, our study covers a period of increasing acceptance of same-sex couples and increasingly liberal attitudes toward people’s choice of family life, much in line with the logic of the second demographic transition arguments (e.g., Lesthaeghe 2014). This is reflected both in public opinion on matters related to sexual minorities and in policy development in relation to marriage and parenthood. In general, developments in the Nordic countries and much of Europe have followed a pattern of increasing acceptance and the legal recognition of same-sex partnership rights, later followed by the recognition of same-sex parental rights as well (cf. Andersson and Noack 2010; for a general discussion, see Cherlin 2009).

In our study, we draw on these developments to relate period trends in same-sex marriage formation and divorce to a few critical junctures (Neyer and Andersson 2008) connected with changes in the legal recognition of same-sex marriages in Sweden: namely, those related to the status of marriage itself and those related to the status of parenthood in same-sex unions. Legal changes related to same-sex parenting in 2003–2005 that paved the way for the truly gender-neutral marriage legislation in 2009 followed the introduction of the registered partnership as the first policy development in 1995. This sequencing of events allows us to study which factors may matter most for the development of same-sex marriage formation and dissolution in the Nordic context. In particular, it allows us to study whether factors related to the symbolic framing of marriage or rather those of practical, legal matters in relation to parenthood may matter the most for the evolution of same-sex marriage trends.

In our study, our primary research question is whether and how different policy changes with regard to same-sex marriage, divorce, and childbearing have affected same-sex family demographic trends over time. We are particularly interested in the change from the recognition of registered partnerships to that of formal same-sex marriages as well as the reforms that increased access to officially recognized parenthood in same-sex couples. We are interested in whether changes to the civil status labeling affected the entry into same-sex unions and whether the increased recognition of same-sex parenting affected childbearing dynamics. Further, we can study whether changes in parental rights affected incentives for same-sex union formation and, consequently, marriage formation. These issues all have important gendered dimensions, in that existing reforms in relation to parenthood have so far primarily facilitated childbearing and childrearing in female same-sex couples.

By addressing these questions, we are better situated to gain insight into several crucial aspects of the policy and gender dimensions of same-sex union dynamics. It also helps us provide insight into the relevance and importance of marriage in general in a context where the normative pressure to enter or exit marriage is relatively weak (e.g., Cherlin 2009; Lesthaeghe 2014).

## Previous Research on Same-Sex Union Dynamics

The legal recognition of same-sex registered partnerships and same-sex marriages adds new dimensions to family-demographic research. It offers new possibilities to study how family dynamics are influenced by the gendered interaction of spouses and how the democratization of marriage is influenced by and influences society at large. For people with any gay or lesbian sexual orientation, it provides new freedoms and opportunities for better legally and socially recognized family life (Badgett 2009; Weston 1991). It may also produce pressures to conform to heterosexually based family norms that are prevalent in society at large (Rydström 2011). In the wake of recent social and legal change, the field of research on same-sex couple relationships has expanded exponentially (Baumle 2018; Moore and Stambolis-Ruhstorfer 2013). The U.S.-based family-demographic literature covers a wide range of topics stretching from the outcomes of partners in same-sex unions (e.g., Bennett 2017; Manning et al. 2016; Rosenfeld 2014) to those of children who grow up in same-sex–headed households (e.g., Biblarz and Stacey 2010; Calzo et al. 2017).

Thanks to the availability of excellent demographic data—and a longer history of legally recognized same-sex unions than in other countries—there is also a relatively vast body of empirical research on same-sex unions in Sweden and other Nordic countries. For example, Andersson et al. (2006) provided the first evidence of same-sex couple dynamics in terms of the formation and divorce of registered partnerships in Norway and Sweden during their first decade of legal existence. Andersson and Noack (2010) extended the same study to also cover Denmark. Wiik et al. (2014) provided an updated study on divorce risks and the role of children in such risks in same-sex marriages in Norway during 1993–2010. These studies demonstrated what can be labelled a “feminization” of same-sex couple dynamics: women in the Nordic region have become much more prone than men to enter same-sex marriages but are also much more likely than men to dissolve their union through divorce. Aldén et al. (2015) provided additional insight into same-sex couple dynamics in Sweden. They studied the earnings and fertility trajectories in same-sex registered partnerships and argued that resource pooling is an important motivation to enter a same-sex registered partnership for men; comparatively, for women, this legal status mainly matters as a basis for family building and parenthood. Other research has focused on further aspects of same-sex family life, such as parental leave use in same-sex and opposite-sex couples (Evertsson and Boye 2017, 2018), retirement dynamics (Kridahl and Kolk 2018), and the educational outcomes of children with lesbian parents (Aldén et al. 2017). Malmquist (2015a, 2015b) offered a set of studies with qualitative research on the different considerations that lesbian couples in Sweden face in relation to family building and family dynamics.

In previous family-demographic research, the issue of divorce and differences in dissolution risks in unions with different composition of the sexes has received much attention (e.g., Andersson et al. 2006; Rosenfeld 2014). Differences in union dissolution risks in which women in same-sex unions show elevated risks have been found repeatedly, in particular for officially recognized unions (e.g., Andersson et al. 2006), with a less clear picture for less stable unions, such as cohabiting relationships (e.g., Lau 2012). Such elevated risks have sometimes been explained through mechanisms related to different forms of social stress in sexual minorities (Doyle and Molix 2015; Meyer 2003). However, previous research has suggested that gender may be a much more decisive factor than sexual-minority status in relation to union-dissolution behavior, with women being more prone than men to initiate divorce both in same-sex unions (e.g., Bennett 2017) and opposite-sex marriages (e.g., Kalmijn and Poortman 2006). Some research has thus suggested that gendered patterns of socialization may matter more for union dynamics than any impact of minority group status (e.g., Orth and Rosenfeld 2018).

In our study, we rely on register data on formal marriages and registered partnerships in Sweden and on demographic methods to analyze these data. Like all quantitative demographic research, our study relies heavily on the quality of available data. In this regard, we are fortunate to have access to excellent longitudinal records of complete civil-status histories of everyone with legal residence in Sweden during a period of almost two decades. This avoids many of the issues related to selective nonresponse or misreporting of events or an individual’s own sex that otherwise create many obstacles for quantitative research on sexual minority family dynamics. For example, available census data from different countries offer only cross-sectional information on people’s living conditions; in addition, they may be biased by sexual minority individuals’ potential unwillingness to disclose sensitive issues related to their sexual orientation or by the marginally huge impact of any misclassification of records on a person’s sex (cf. Black et al. 2000; Cortina and Festy 2014; Festy 2007). The latter also holds for sample surveys that are not explicitly geared toward individuals with a sexual minority belonging (e.g., Reczek et al. 2017; Régnier-Loilier 2018; Sullins 2017). Other surveys may be directed toward GLBT individuals but build on sampling methods that make it difficult to judge what population the surveyed participants actually represent.

Clearly, relying on official records of civil-status changes also has disadvantages. For example, such records provide little information on any subjective dimensions of couples’ lives. They are also unable to delineate other dimensions of union trajectories, such as those related to nonmarital cohabitation and dating or “living apart together” relationships. We also have no data on issues such as the sexual orientation or gender identity of partnered and nonpartnered individuals in Sweden. In what follows, we proceed with a presentation of the Swedish context of same-sex registered partnerships and marriages, and then our data and methods, before presenting our empirical results in terms of same-sex marriage formation, childbearing, and divorce.

## The Introduction of Same-Sex Marriages in Sweden

To address our research question of how the changing policy landscape of Sweden has been related to family-demographic trends for same-sex couples, we briefly introduce the evolution of policies in relation to same-sex couples following the introduction of legislation for registered partnerships in Sweden in 1995. During the late 1980s and early 1990s, the Nordic countries were forerunners in granting legal recognition to partners of the same sex. Denmark initiated this development in 1989 by introducing an entirely new civil status, *registered partnership*, for this purpose. Norway introduced the registered partnership in 1993; Sweden and Iceland followed suit in 1995 and 1996, and Finland introduced it in 2002. The new civil status was different in name but otherwise similar in contents to that of (heterosexual) marriage. With a few but important exceptions, registered partnership conferred the same legal rights and duties as marriage provides to opposite-sex couples and therefore amounted to a de facto same-sex marriage. In the Nordic countries, rights related to marriage are not very extensive: most social rights hold independently of a person’s marital or family status (e.g., taxation, pension benefits, and access to health care and social insurance). In Sweden, the exceptions compared with heterosexual marriages consisted of one or more of the following issues: the opportunity to jointly adopt a child, access to medically assisted insemination, the forms of how to solemnize the partnership, and requirements of legal residency in the country before entering a partnership—or as Jens Rydström (2011) referred to them, the three C’s related to *children*, the *church*, and *citizenship*. These exceptions were questioned, however, and many of them were abolished during subsequent years. In 2003, registered partners in Sweden were allowed to jointly adopt a child; in 2005, medically assisted insemination was made available to women in same-sex cohabiting relationships. The legal changes in 2003 were particularly important: this was the first time the law acknowledged that a child can have two legal parents of the same sex (Malmquist 2015a). Crucially, the access to adoption was restricted to partners in a registered partnership or marriage. (Surrogacy motherhood has been and remains prohibited in Sweden.) From the outset, the procedures for dissolving a registered partnership were the same as for opposite-sex spouses. In our study, we refer to both registered partnerships and formal marriages as “same-sex marriage” because little else other than the union’s label changed with its full legal recognition in 2009.

In 2009, Sweden completed its process of granting same-sex couples the same rights to marriage as those granted to couples of opposite sexes by adopting a fully gender-neutral marriage legislation. (Formal same-sex marriage was introduced the same year in Norway; it was introduced in Iceland in 2010, in Denmark in 2012, and in Finland in 2017.) Simultaneously with the new legislation in 2009, the Church of Sweden voted to accept the solemnization of same-sex marriages in Swedish churches; marriages can also be solemnized in civil ceremonies through local municipalities without involvement of the church. No new registered partnerships could be formed subsequent to the introduction of the new legislation in May 2009. Couples who had already entered a registered partnership could, however, retain their civil-status label as registered partners if they wanted to. Alternatively, they could choose to convert their civil status to marriage, as mainly a symbolic act given that there were no longer any legal differences attached to the statuses of an already registered partnership and a same-sex marriage.

## Data, Methods, and Study Design

Our analyses are based on Swedish register data, and in particular the civil-status register that covers information on all registered changes in the marital status of each individual living in Sweden. Statistics Sweden produces statistics on the number of women and men in different family types and on changes in the civil status of women and men with residence in Sweden. During our study period, Sweden lacked a register on residence by unique dwelling units, which makes it impossible to study cohabiting unions. However, even with the presence of such registers, same-sex cohabitants would be very hard to numerate correctly based on administrative data sources (Festy 2007; Kreider and Lofquist 2015).

Our statistical analyses are based on Swedish-born individuals who were under the risk of experiencing any civil status change during 1995–2012, and the civil-status changes experienced by these individuals. To avoid considering how migration and family formation interrelate, we choose to exclude foreign-born individuals from our study population. (A substantial fraction of spouses in male same-sex couples are foreign-born; see Andersson et al. 2006).^1^ We control for any previous civil-status histories during the 1970s onward. Our analyses include both women and men who may form or dissolve same-sex and opposite-sex marriages. We study the rates of only first marriage formation and the dissolution of first marriages, given the very small number of same-sex marriages in higher-order marriages (only 3% of new spouses in same-sex marriages had previously been married to a partner of the same sex). However, our analyses include unmarried women and men at risk of first same-sex marriage formation who previously had been in an opposite-sex marriage (about 10% of new spouses in same-sex marriages had been previously married in an opposite-sex marriage).

Our civil-status records are linked to childbearing records through Swedish birth registers. These registers cover biological parenthood, and as such, every child is supposed to be registered with the biological father and mother. In practice, close to 99% of children in Sweden are indeed registered to a (presumably) biological mother and father; however, in the case of children born in same-sex relationships, these fractions are much lower (see the Results section). In this manner, the multigenerational register contains information on biological parenthood if the child and the parent(s) are registered as residents of Sweden. Previous research has shown that significant portions of individuals in same-sex marriages have children from previous opposite-sex unions and that this holds in particular for women (Andersson et al. 2006).

Our analyses of same-sex and opposite-sex marriages cover all new unions formed by Swedish-born individuals aged 16–49 during 1995–2012. For our event-history analyses of first-marriage formation, individuals are under risk from age 16 or January 1, 1995, until the event of marriage or censoring due to emigration, age 50, death, or the end of 2012, whichever comes first. The analyses of opposite-sex marriage formation censor at same-sex marriage formation, and vice versa. Individuals who divorce from an opposite-sex marriage reenter the study upon divorce as being under risk again of first same-sex marriage formation. We include a time-varying covariate to identify these individuals. With the introduction of the new gender-neutral marriage legislation in 2009, a minority of registered partnerships were converted into formal marriages. Slightly more than one-quarter of couples took advantage of this opportunity during our follow-up until 2012; 10% did so in the first year when the law was introduced. Our models track such unions from the start of their registered partnership, but we do not treat them as forming a new union when converting their partnership to a marriage.

Similarly, our analyses of divorce risks consist of all individuals who formed a registered partnership or marriage in 1995 and later; individuals are under risk until a divorce occurs or until censoring due to widowhood, emigration, own death, reaching age 50, or the end of 2012, whichever comes first. Individuals who transformed their registered partnership into a formal marriage in 2009 or later remain in the study population, with the characteristics carried over from the partnership union.

We include a covariate with annual period dummy variables in our hazard regression models to examine how the relative propensity of marriage formation and divorce changed over all calendar years since 1995. In our models, we use 2002 as a reference year because in that year, the absolute numbers of newly formed male and female same-sex marriages were approximately the same. We include a number of demographic covariates: for age (as dummy variables in mainly three-year age groups from ages 16 to 49) and parity (0, 1, 2, and 3 or more children); and, in the divorce models, for premarital childbearing (yes/no) and the duration since marriage formation (as year dummy variables at different durations from 0 to 15 years since marriage formation). Additionally, for same-sex marriage formation, we apply a time-varying covariate on whether an individual previously experienced being in an opposite-sex marriage (yes/no). Further, we present descriptive statistics to study the prevalence of previous childbearing at the time of marriage formation and circumstances for continued childbearing during the first five years following marriage formation.

The purpose of our analyses is to follow how period trends in same-sex marriage formation and divorce have evolved over time. In particular, we aim at relating them to two critical junctures in terms of the legal status of same-sex marriages in Sweden: one related to the liberalization of the rules connected to parenthood in same-sex marriages that occurred in 2003 and 2005, respectively, and another connected to the change in the status of marriage in terms of solemnization procedures and the actual labeling of “marriage” that occurred in 2009. Both changes may have had an impact on intensities in marriage formation and divorce, and the impact may have been different for women and men. In the first case, it may differ if women and men have different choices to respond to the new possibilities of parenthood in same-sex unions. In the second case, it may differ if women and men attach different values to the label of marriage and the status of being married. For some, marriage may appear as a more attractive and inclusive label that perhaps signals a more committed union type than that of a partnership. If this is the case, the policy change in 2009 could have been associated with higher propensities to form new marriages and perhaps lower risks of divorce.

In terms of population numbers, our study covers longitudinal information on 2,142,905 men and 1,893,518 women born in Sweden, of which 4,230 women and 2,444 men formed a same-sex marriage when they were younger than 50 during our study period. The data cover the entire populations under risk. In the next section, we present the relative risks of marriage formation and divorce, by calendar year, in diagrams. Tabulated relative risks and the confidence intervals for those risks are shown in the online appendix (Tables A1–A4). These tabulations also contain model extensions where we additionally control for the role of educational attainment and study enrollment in marriage and divorce behavior. Our study is not based on any methods of population sampling. However, because of relatively few observations in some calendar years, several relative risks are attached to rather large confidence intervals. In Tables A5 and A6 in the online appendix, we therefore provide relative risks by calendar years divided into broader categories, with larger numbers of observations in each category. This assures that all statements on differences in risks over time are true when standard statistical tests are applied.

## Results

### Changes in Same-Sex Marriage Formation, 1995–2012

**Fig. 1:**
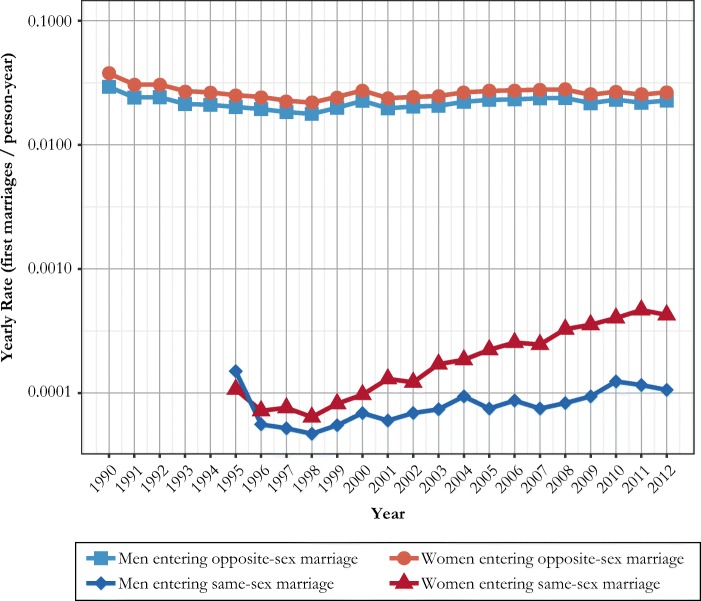
Annual rate of first same-sex and opposite-sex marriage formation for Swedish-born never-married men and women at ages 16–49. Number of marriages divided by never-married population at risk, 1990–2012. *Source:* Swedish register data, authors’ own calculations.

We begin by showing how the crude rates of same-sex marriage formation changed since the introduction of the registered partnership in 1995 (Fig. 1). We define first same-sex marriages of women and men at ages 16–49 as our *occurrences*, and the unmarried population at ages 16–49 as our *exposures*. We also present the corresponding rates of opposite-sex marriage formation as a comparison; because of different magnitudes in rates, these differences are shown on a logarithmic scale. There was an initial spike in rates of same-sex marriage formation just after the introduction of the registered partnership in 1995, reflecting pent-up demand. This spike was more marked for men than for women. However, during all calendar years after 1995, Swedish-born women had consistently higher rates of same-sex marriage formation than men.

Men’s rates of same-sex marriage formation clearly increased over time, with crude rates doubling from 0.0056% to 0.0106% per year between 1996 and 2012, but the crude rates for women increased much more rapidly (Fig. 1). The probability of a woman entering a same-sex marriage in a given year increased sixfold, from 0.0072% to 0.0426%, between 1996 and 2012. These increases took place during a period in which the rates of opposite-sex marriage formation also increased. A closer inspection reveals that the marriage rates started to increase for all three types of marriages around 1999. The increase in heterosexual marriage formation based on our logarithmic representation does not appear strong, but it amounts to a relative increase by 20% in marriage rates between 1998 and 2012.

**Fig. 2:**
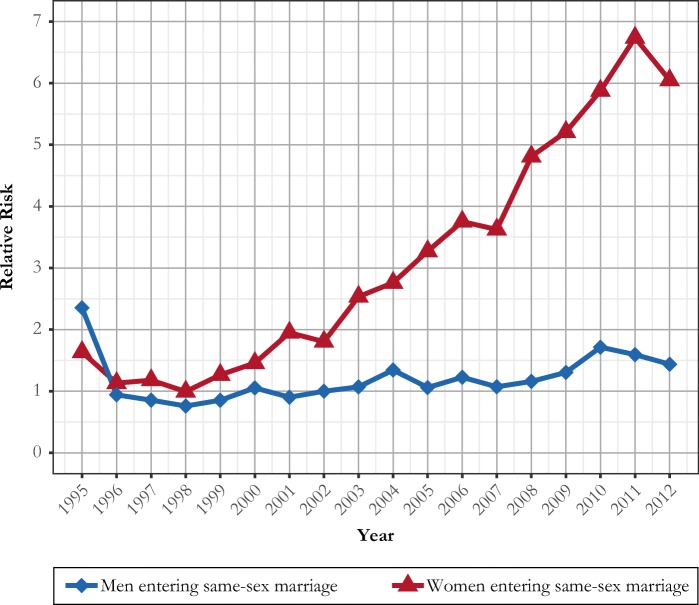
Relative risks of first same-sex marriage formation in Sweden, by calendar year and sex, 1995–2012. Risks relative to that of men in 2002. Risks are standardized for age group, parity, and the experience of any previous opposite-sex marriage. Source: Swedish register data, authors’ own calculations.

We further analyze the risk of first same-sex marriage formation by applying event-history analyses in which we standardize for underlying changes in the composition over demographic covariates over time (Fig. 2). These results corroborate the results from our presentation of crude rates. We find a very rapid increase in the risk of same-sex marriage formation over time, particularly for women. At the beginning of the 2010s, women had a relative risk of same-sex marriage formation about three to four times as high as that of men. Interestingly, it appears that the introduction of formal same-sex marriage legislation in 2009 did not have any noticeable impact on the rates of new same-sex marriages. Both for men and women, we observe an absence of trend change in the rates of new same-sex marriage formation around 2009 (although a number of women and men chose to convert their registered partnership into a formal marriage; see the previous section). After 2010–2011, the increase appears to have leveled-off, and the risk even declined modestly in our last year of observation. In contrast, the granting of adoption rights to same-sex couples in 2003 and the new rights to medically assisted reproduction in 2005 may have fueled the trend of increasing rates of female same-sex marriage formation. The long-term trend in the relative risks of same-sex marriage formation of women appears to accelerate in 2003; subsequent increases appear in parallel to increasing rates of childbearing in female same-sex marriages (see the following section). Our results are controlled for the impact of compositional changes over our other variables at hand, including the very low risks of same-sex marriage formation for nonmarried parents, most of whom live in a (not-registered) cohabiting heterosexual relationship (Table A1, online appendix).

The importance of considering both absolute numbers of marriages and the demographic rates of marriage formation is underlined by the different age profiles of opposite-sex, female same-sex, and male same-sex marriage formation. Peak intensities of first-marriage formation occur at younger ages for opposite-sex than for same-sex spouses. Additionally, female same-sex marriages are formed earlier than marital unions of two men (see the age-specific rates in Fig. A1, online appendix). Given that same-sex marriage formation of men occurs much later in life, men also spend much more time “at risk” of getting married, which reduces their rates of marriage formation. The faster motion of women into marriage translates into higher rates of same-sex marriage formation.

For opposite-sex couples, marriage has increasingly become connected to parenthood, and recent increases in the formation of opposite-sex marriages in Sweden are linked to increased rates of marriage formation of parents, as shown in Fig. A2 in the online appendix. This development also motivates us to look deeper into the role of childbearing and parenthood in relation to same-sex marriage formation.

### Childbearing in Same-Sex Marriages, 1995–2012

**Fig. 3:**
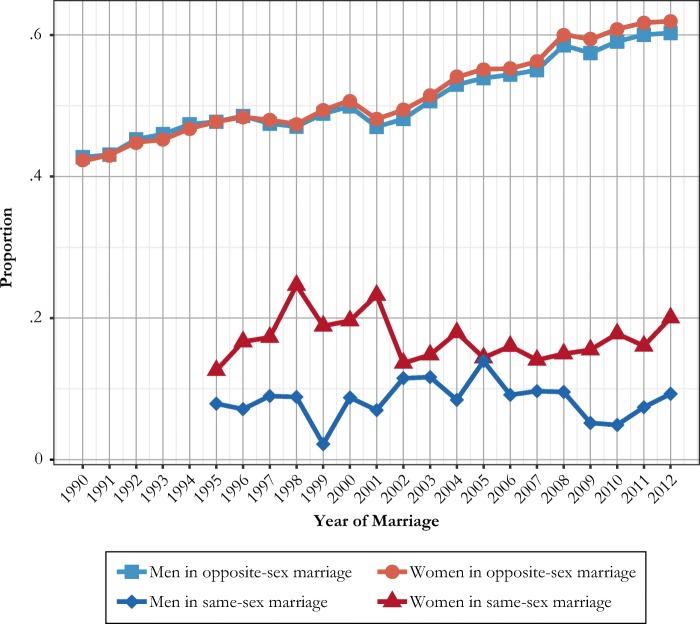
Annual proportions of women and men who enter first same-sex and opposite-sex marriage who are parents at the time of marriage formation, Swedish-born men and women at ages 16–49. *Source:* Swedish register data, authors’ own calculations.

Consequently, we turn to the analysis of childbearing in relation to same-sex marriage. As described in the Data section, our results refer to biological children of an individual as registered by the authorities, unless noted otherwise. We begin by showing the prevalence of premarital childbearing at the time of marriage formation, which in many cases reflects childbearing from a previous heterosexual relationship. Figure 3 shows trends for childbearing before same-sex marriage formation relative to childbearing before opposite-sex marriage formation. A stable proportion of women (a little less than 20%) and men (a little less than 10%) who enter same-sex marriage have children at that time. For opposite-sex marriages, we find an increasing share of women (more than 50%) with premarital children at entry into marriage.

**Fig. 4:**
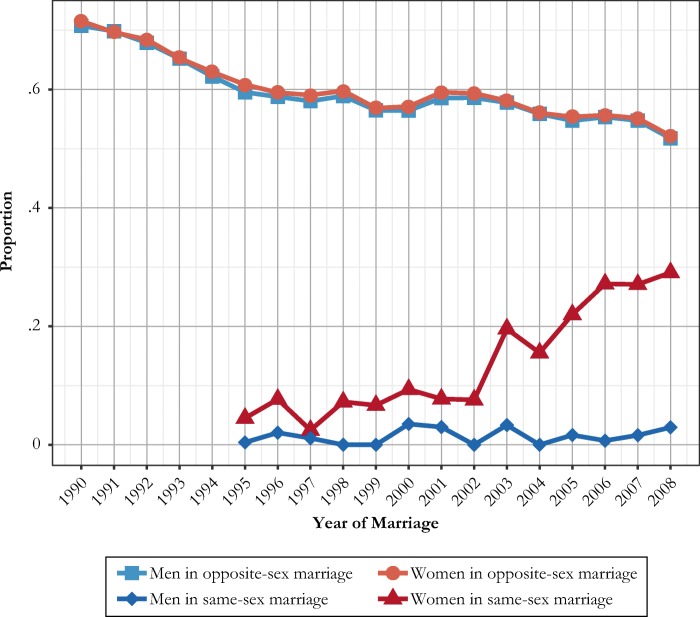
Annual proportions of women and men in first same-sex and opposite-sex marriages who had a child within five years of marriage formation, Swedish-born men and women at ages 16–49. *Source:* Swedish register data, authors’ own calculations.

Another and more recent phenomenon is that of increasing childbearing *after* same-sex marriage formation (Fig. 4), which provides evidence for the increasingly important link between marriage and parenthood for lesbian partners in particular. Unlike many cases of premarital childbearing, these indeed occur in relation to the other same-sex spouse. Figure 4 displays the levels of individual childbearing after marriage formation, by type of marriage and marriage cohort. We observe all spouses during their first five years of marriage, which makes our analyses end in 2008. The figure shows that although childbearing was relatively uncommon after same-sex marriage formation in the 1990s, it began to increase substantially for women after the legal changes implemented in the early 2000s. Men have consistently had very low proportions of marital childbearing (Fig. 4). In the last marriage cohorts for which we have data during a five-year follow-up, some 20% to 30% of individual women in same-sex marriages had a child during the follow-up. When these numbers are aggregated to the couple level, these fractions become much larger.

Our childbearing data pertain to records of own biological children, as registered by Swedish authorities. Still, by linking spouses to each other and biological and adopted children to their registered parents, we can derive additional albeit still partial information on further aspects of parenting in same-sex unions. Evidently, the opportunities to become a parent are much greater for women than for men in same-sex marriages. This holds for possibilities for own biological motherhood by means of assisted reproduction (Rozental and Malmquist 2015) as well as opportunities for second-parent adoption (Malmquist 2015b). In addition, the childbearing history of just one spouse in a same-sex marriage does not cover what happens at the couple level. To study this in better detail, we further examine whether any or both partners in a new marriage had another registered child within the first five years of marriage. This analysis (see Fig. A3, online appendix) shows that almost 50% of all female same-sex marriages formed in 2008 produced at least one child during the first five years of marriage. In 7% of female same-sex marriages formed in that year, both women gave birth to a child. The level of childbearing in female same-sex marriages is thus comparable to current levels in opposite-sex marriages even though the patterns of premarital childbearing differ. However, the dynamics do not simply mirror what happens in opposite-sex marriages. For same-sex marriages, it is essential to analyze both spouses simultaneously to assess the combined fertility because births can potentially also be spaced much closer than in opposite-sex unions.

Adoptions also matter for same-sex parenting. Since 2003, adoption in same-sex couples has been available to couples in a registered partnership or marriage (Malmquist 2015b). In Sweden, there is generally very little domestic adoption (Barclay 2015; Johansson 2011), and same-sex spouses often face difficulties when donor countries decline same-sex adoptive parents. These restrictions are most severe for gay couples. However, since 2003, the number of second-parent adoptions has increased, in which the spouse of one partner adopts the biological (or adopted) child of the other spouse (Malmquist 2015b). In 82% of cases, these adoptions take place during the first year of the child’s life. The practice is concentrated to female same-sex unions and requires the formation of a registered partnership or marriage. Further, in 2005, same-sex couples in Sweden were granted the legal right to medically assisted reproduction at low cost and on the same terms as opposite-sex couples, which has been widely accessible to lesbian spouses (Rozental and Malmquist 2015). (These rights also hold for cohabiting nonmarried couples, but we currently lack data on any coparenting in same-sex cohabiting unions.) The latter development is partly reflected in Fig. 4. In these cases, the partner can be granted automatic parental rights without the process required for second-parent adoption.

**Fig. 5:**
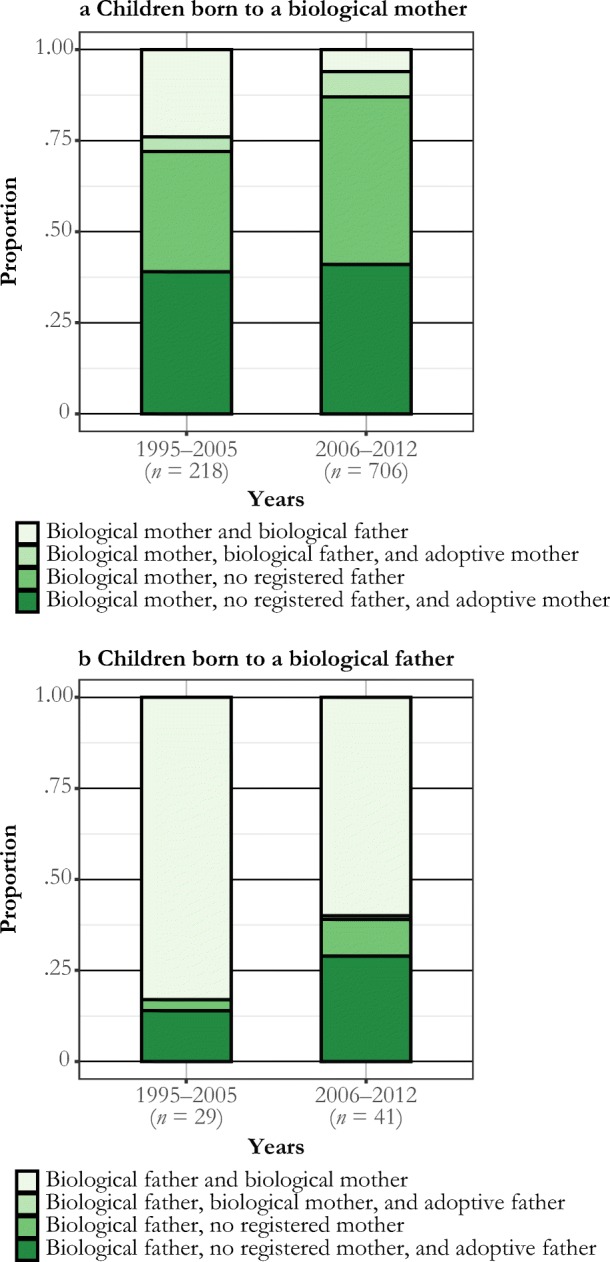
Proportions of children born to a biological mother (panel a) and father (panel b) in a same-sex marriage in Sweden with different constellations of registered parents. Data for the first biological child to a Swedish-born mother or father within five years of same-sex marriage formation. Children born before and after the availability of assisted reproduction in 2005. *Source:* Swedish register data, authors’ own calculations.

In additional analyses, we produce data on the family situation of children born to mothers and fathers in registered partnerships and same-sex marriages (Fig. 5). As before, these data refer to the (registered) biological children of a Swedish-born mother (panel a of Fig. 5) or father (panel b of Fig. 5) during the first five years of a same-sex marriage. Most children were born in a female same-sex marriage. The data show that for a vast majority of these children, the father is unknown in the register. In some of these cases, paternal unknown status is due to assisted reproduction taking place outside Sweden—often in Denmark, where assisted reproduction is offered with less formal requirements in terms of, for example, the registration of a biological father (Malmquist 2015a). In 40% to 50% of cases, the child also has a registered adoptive mother. Figure 5 distinguishes between children born during 1995–2005 and those born in 2006–2012—that is, those born before and after the legal reforms related to parenting in same-sex unions. It was much less common for children born in the latter subperiod to have a registered biological father.

For the much fewer children born to men in a same-sex marriage, the picture is different (panel b of Fig. 5). In the majority of cases, the child also has a registered biological mother in Sweden. Our register data contain no further information on legal custodians of the children involved.

### Changes in Divorce Risks in Same-Sex Marriages, 1995–2012

**Fig. 6:**
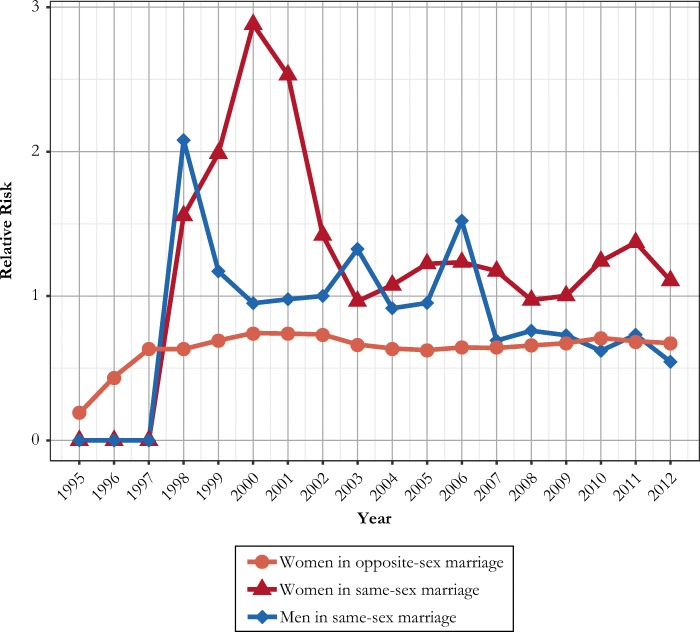
Relative risks of divorce in Sweden, by calendar year and type of marriage, 1995–2012. Risks relative to that of men in same-sex marriages in 2002. Risks are standardized for duration of marriage, age group, premarital childbearing, and parity. The comparison is based on marriages formed during 1995–2012. *Source:* Swedish register data, authors’ own calculations.

Following our analyses of entry into marital unions, we also present information on changes in the dissolution of same-sex unions, with comparisons to trends in divorce risk for opposite-sex marriages (Fig. 6). Divorce risks appear somewhat lower at the end of the study period than during earlier years. People in same-sex marriages have higher divorce risk than those in opposite-sex marriages, although the differences have declined over time, suggesting some convergence of the dynamics in same-sex and opposite-sex marriages.

We also find substantive differences in the divorce risks of women and men in same-sex marriages. In the late 1990s and early 2000s, women had very high divorce risks, much higher on average than for men in same-sex marriages and for people in opposite-sex marriages. For a few years, women in same-sex unions had divorce rates that were more than three times as high as those for people in opposite-sex unions. These differences declined somewhat over time, but female same-sex marriages still are more likely to end in divorce than male same-sex and opposite-sex marriages. The divorce risk for men in same-sex marriages also declined, and toward the end of our study period, it approached the divorce risk of spouses in opposite-sex marriages. Compared with people in other unions, women in same-sex unions have a divorce rate that is nearly twice as high. This pattern appears to have stabilized with no major changes across years, perhaps indicative of a more stable long-term pattern. We find no evidence of any major change in divorce risks after the introduction of formal same-sex marriage legislation in 2009, although our short follow-up period makes such changes difficult to identify. The divorce risks in opposite-sex marriages were largely stable during our study period. The full results for the models in Fig. 6 are available in Appendix Table A3 in the online appendix.

**Fig. 7:**
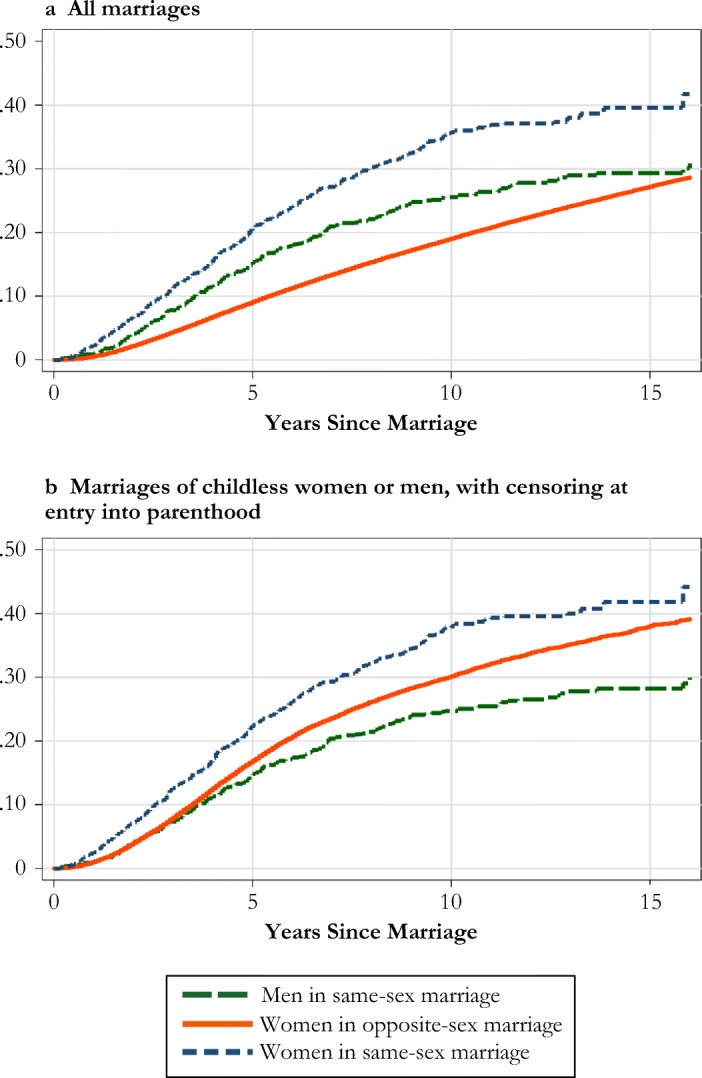
Cumulative proportion divorced, by type of marriage in Sweden, 1995–2012, for all marriages (panel a) and for marriages of childless women or men with censoring at entry into parenthood (panel b). *Source:* Swedish register data, authors’ own calculations.

In panel a of Fig. 7, we graph the cumulative incidence of divorce for women and men in same-sex marriages compared with people in opposite-sex marriages. Unlike our event-history models, these models do not standardize for any further demographic variables (such as age, calendar period, and parity). However, the description is based on the same study design as before and confirms the picture from our previous analyses. The tendency to divorce is highest among same-sex married women, followed by same-sex married men and opposite-sex married spouses. Same-sex marriages seem to be particularly unstable at very brief periods after marriage formation, compared with the dynamics in opposite-sex marriages. After 15 years of follow-up, approximately 30% of male same-sex marriages and opposite-sex marriages ended in divorce, and 40% of female same-sex marriages ended that way.

Panel b of Fig. 7 provides further insight into these dynamics by restricting the study to cover only childless unions. In this case, we present the cumulative incidence of divorce by using a life table approach in which we censor individuals when they become parents. We find that although the pattern for same-sex marriages changed relatively little compared with the patterns in panel a (parenting is still less common in these marriages), childless opposite-sex marriages are under significantly higher risk of divorce than when all marriages are considered. Among childless marriages, same-sex marriages of men have the lowest probability of ending in divorce.

## Discussion

Our study demonstrates a number of new developments as well as continuities in the dynamics of same-sex marriage and divorce in Sweden during 1995–2012. We observe a continuous increase over time in the prevalence of same-sex registered partnerships and marriages, albeit from low levels. This holds for female same-sex marriages in particular and occurred in parallel with simultaneous increases in the propensity of Swedish women and men to form opposite-sex marriages (cf. Andersson and Kolk 2015; Ohlsson-Wijk 2011). In the final years of our study period, this increase seems to have leveled-off, and this trend change appears to have happened in tandem with a similar leveling-off in the trends of opposite-sex marriage formation (Andersson and Kolk 2015). To some extent, the positive correlation of same-sex and opposite-sex marriage trends may signal a normalization of same-sex marriage dynamics, such that the different contextual factors that may make marriage more or less attractive affect same-sex and opposite-sex couples similarly.

Our study also confirms previous observations from Sweden and other Nordic countries (Andersson and Noack 2010; Andersson et al. 2006) of crossovers in the same-sex marriage formation of women and men such that this family type has become increasingly female-dominated. Similar findings have been observed in other countries (Andersson et al. 2006; Chamie and Mirkin 2011; Cortina et al. 2012; Ross et al. 2011). Our extended follow-up period and the application of improved methods of analysis allow us to demonstrate excess risks of same-sex marriage formation of women versus men that may not have been observed before, although statistics from the United States also suggest a strong overrepresentation of female same-sex marriages in some states (Chamie and Mirkin 2011).

Another recent phenomenon that deserves further attention is the increasing prevalence of childbearing in female same-sex marriages (Aldén et al. 2015; Evertsson and Boye 2018; Wiik et al. 2014). We demonstrate that about one-half of female same-sex registered partnerships or marriages at the end of our study period were accompanied by the subsequent childbearing of at least one of the two partners involved. Other spouses bring children from a previous relationship to the new marriage. The empirical evidence suggests that recent policy changes in relation to parental rights were crucial in driving the trends of increasing marriage formation of lesbian women: only the registered partnership or marriage provides the necessary means to assure a spectrum of parental rights. Clearly, the possibilities for gay couples to realize any childbearing desires are much more limited than those for women in same-sex couples.

Our results for the divorce risks in same-sex marriages also show a few changes as well as continuities in their developments over time. We find some evidence of a convergence of divorce risk levels in opposite- and same-sex marriages. This is particularly pronounced for male same-sex marriages, which show divorce risks that are entirely similar to those of opposite-sex marriages at the end of the study period. The elevated divorce risks among same-sex marriages reported for Sweden in earlier research (Andersson et al. 2006) are no longer as pronounced as they were in the 1990s. For childless marriages, the differences between opposite- and same-sex marriages are even smaller; male same-sex marriages show substantively lower dissolution rates than childless opposite-sex marriages. Perhaps the normalization of same-sex marriages in Sweden has contributed to making the demographic behavior of same-sex couples increasingly similar to that of their heterosexual peers.

Still, the averages of statistics for same-sex couples of women and men are partly misleading because the differences in divorce risks between female and male same-sex marriages are much larger than the differences between opposite-sex marriages and the combined population of same-sex couples. Our study confirms that the previously reported pattern of elevated divorce risks in couples of two women (Andersson et al. 2006) remains intact. Meanwhile, similar patterns of elevated divorce or union dissolution risks of couples of two women compared with those of two men have been reported in most other contexts where data on legally recognized same-sex unions are available, including Norway (Wiik et al. 2014); Denmark (Andersson and Noack 2010); Belgium, the Netherlands, and Spain (Chamie and Mirkin 2011); England and Wales (Ross et al. 2011); and the United States (e.g., Ketcham and Bennett 2016; Rosenfeld 2014). For further cross-country surveys, see Bennett (2017) and Marteau (2017).

A key contribution of our study is that it is based on longitudinal and reliable demographic data in terms of registered civil-status changes during a period of almost two decades and the application of appropriate longitudinal demographic methods to these data. In terms of methods, we demonstrate the importance of relying on demographic rates and life table techniques in order to properly study changes in levels of marriage formation and divorce over time. Such an approach is important when studying the rates of opposite-sex and same-sex marriage formation because the age profiles of different types of marriage formation, along with the corresponding exposure times under risk of getting married, differ substantially (cf. Fig. A1, online appendix).

In our analyses, we consider and control for a set of purely demographic characteristics. Other factors matter as well, including educational characteristics, with same-sex spouses often having higher educational attainment than opposite-sex spouses (Andersson et al. 2006). As a robustness check, we estimate trends in marriage formation and divorce where we also adjust for people’s educational attainment (Tables A2 and A4, online appendix). The period trends in marriage formation and dissolution appear very similar when those controls are added. The educational gradient in marriage (positive) and divorce (negative) is very similar for men and women who enter and leave same- and opposite-sex marriages.

Beyond presenting an accurate picture of demographic change among same-sex couples in Sweden, the goal of our study is to relate family-demographic change to a set of policy interventions during the same period. In particular, we are interested in whether reforms related to the legal recognition of parental rights in same-sex unions or those related to the status and label of marriage as such may have mattered the most for the dynamics of same-sex marriage formation and divorce. Our evidence suggests that the former factor appears to matter greatly, while the latter factor largely goes unnoticed in terms of any relation to same-sex family-demographic trends. It also becomes clear that the impact of changing policy and legal frameworks seems much more pronounced in the family dynamics of female couples than in couples of two men. The policy change in 2003—the first time that parental rights of two persons of the same sex were explicitly acknowledged in the law—indeed appears to have been crucial. It supported the practice of second-parent adoption, which like other procedures for adoption in Sweden, requires that the two partners are married (or in a registered partnership). The subsequent policy change in 2005 provided additional regulation and procedures for parenthood that offered additional incentives for prospective parents to get married. Following the introduction of these reforms, the rates of female same-sex marriage formation began its long-term increase; the divorce risks in couples of two women were also lower than before the reforms. Because parental rights in Sweden are otherwise only weakly related to parents’ marital status, the legal status attached to marriage often matters more for same-sex spouses than it does for women and men in opposite-sex unions. We speculate that the increasing prevalence of children in female same-sex marriages may continue making the demographic dynamics of male and female same-sex couples very different in the future. Future policy changes may alter these relations: in 2016, assisted reproduction was made available in Sweden also to nonmarried, single women. In contrast, there are currently no legal possibilities for surrogacy motherhood.

We find that in contrast to the first two policy interventions, the introduction of gender-neutral marriage legislation and formal same-sex marriages in Sweden in 2009 does not appear to have affected trends in same-sex marriage dynamics to any visible extent. A further inspection of our data also reveals that relatively few couples reacted to the possibility to transform their existing registered partnership to a marriage during the years when no new partnerships could be formed (about one-quarter of previous couples during our follow-up in 2009–2012). The weak effect of formal same-sex marriage legislation suggests that when the underlying legal differences between registered partnership and formal marriage are minor (Andersson and Noack 2010), symbolic changes in the labeling of unions appear relatively unimportant in individual decisions on whether to marry. This situation may be very different from that in, for example, the United States, where marriage is ascribed a much more normative and ideological status than is the case in most countries in Europe (Cherlin 2009).

Our study underlines the striking pattern of what we may label the feminization of same-sex marriage dynamics. Women are much more prone than men to both enter and dissolve same-sex marriages. To some extent, these gender-specific differences relate to differences in behavior that can be observed for women and men in opposite-sex couples, with women often initiating marriage but also being more likely than men to initiate divorce (e.g., Hewitt et al. 2006; Kalmijn and Poortman 2006). Divorce may sometimes be traumatic, and not less so if partners are exposed to the additional burden of minority stress (Balsam et al. 2017). Perhaps divorce is stimulated by women often being more sensitive than men to different aspects of relationship quality (e.g., Shieh 2016). In heterosexual couples, women often report that they are less satisfied than men with their unions (Wiik et al. 2012). Our research on dissolution risks in same-sex marriages may be contrasted with a recent body of literature from the United States that instead highlights other dimensions of couple dynamics than those we cover, such as dating and “living apart together” relationships. These studies suggest that gay men may instead be more prone than lesbians to initiate and terminate dating and couple formation (Joyner et al. 2017; Orth and Rosenfeld 2018). An interesting feature of this literature is that it also highlights the similarities in behavior of women and men in same- and opposite-sex couples as well as the importance of childhood socialization in creating gender-specific behaviors in relation to couple dynamics (Orth and Rosenfeld 2018).

We conclude with some reflections on research on marriage, parenthood, and divorce in same-sex marriages. In a range of societies, marriage and divorce are considered basic human rights, whereas in other contexts, they are more controversial issues. Cherlin (2009) highlighted that in most countries in Europe, issues of parenting in same-sex couples have tended to be more controversial than those related to granting same-sex couples the right to legalize their unions. Registered partnerships have been introduced in many more countries in Europe than has the adoption of children to couples in same-sex unions (Davis 2013; see also Waaldijk et al. 2017). This story also fits the Swedish case, where registered partnership paved the way for the legal recognition of parenting in same-sex couples, which in turn made the gender-neutral marriage legislation something of a *fait accompli*. In the United States, the situation has been somewhat different, with adoption rights often being less controversial than other aspects of the democratization of marriage to also include couples of the same sex (Cherlin 2009). The right to divorce is rarely contested, but the issue of divorce has sometimes been used as an argument for or against same-sex marriages (e.g., Sears 2015). Divorce levels in general differ across contexts without much bearing for the status of marriage in those contexts (Andersson and Philipov 2002; Andersson et al. 2017). To put our results on divorce risks in international context, we note that the elevated divorce risks in female same-sex marriages in Sweden are similar to the risks observed for spouses in opposite-sex marriages in the United States.

Sweden has often been in the forefront of family and gender change and the interaction of those developments (e.g., Goldscheider et al. 2015). In our current contribution, we highlight an increasing feminization of same-sex family dynamics, the increasing prevalence of motherhood in those dynamics, and a process in which same-sex union dynamics often appear in sync with those of different-sex marriages. We recommend that future research should continue following the family demographic trends of same-sex couples in Sweden in relation to the ever-changing landscape of legal provisions for same-sex parenthood, particularly the effect of these changes on opportunities for parenthood among men and women in same-sex couples.

## Electronic supplementary material

ESM 1 (PDF 680 kb)

## Footnotes

1 During our study period, 6% of new same-sex marriages consisted of two foreign-born individuals, 25% of couples had one foreign-born person, and 69% of couples involved two Swedish-born spouses. Our analyses cover all individuals born in Sweden, regardless of the country of birth of any spouse.
