# Supplementary material for: Two Decades of Same-Sex Marriage in Sweden: A Demographic Account of Developments in Marriage, Childbearing, and Divorce
Source: Demography. 2020 Jan 9;57(1):147–69. doi: 10.1007/s13524-019-00847-6 (PMC7052034; doi:10.1007/s13524-019-00847-6)
Supplement: Supplementary file 1 — (PDF 680 kb) [file 13524_2019_847_MOESM1_ESM.pdf]

Table A1: Event history model on the risk of first same-sex marriage formation, men and women in Sweden, including the interaction of calendar period and sex (risks relative to that of men in 2002)

	Relative risks	95% C. I.	
Period - Same sex men			
1995	2.35	1.87	2.96
1996	0.94	0.72	1.24
1997	0.86	0.65	1.13
1998	0.76	0.57	1.01
1999	0.85	0.65	1.13
2000	1.05	0.81	1.37
2001	0.90	0.69	1.18
2002	1		
2003	1.07	0.83	1.39
2004	1.34	1.05	1.72
2005	1.06	0.82	1.37
2006	1.23	0.96	1.57
2007	1.07	0.83	1.39
2008	1.16	0.90	1.49
2009	1.31	1.02	1.67
2010	1.71	1.36	2.16
2011	1.59	1.26	2.02
2012	1.44	1.13	1.83
Period - Same sex women			
1995	1.64	1.25	2.14
1996	1.13	0.85	1.51
1997	1.18	0.89	1.57
1998	0.99	0.74	1.34
1999	1.27	0.96	1.67
2000	1.46	1.12	1.91
2001	1.95	1.52	2.50
2002	1.80	1.40	2.32
2003	2.53	2.00	3.21
2004	2.76	2.19	3.48
2005	3.27	2.62	4.10
2006	3.75	3.01	4.67
2007	3.62	2.91	4.52
2008	4.81	3.89	5.95
2009	5.21	4.22	6.43
2010	5.88	4.77	7.23
2011	6.74	5.49	8.27
2012	6.05	4.92	7.44
Parity			
0	1		
1	0.14	0.12	0.15

	2	0.04	0.04	0.05
	3+	0.04	0.03	0.05
Age				
	16-18	0.02	0.01	0.03
	19-21	0.11	0.09	0.14
	22-24	0.44	0.39	0.50
	25-27	1		
	28-30	1.86	1.68	2.06
	31-33	2.85	2.58	3.15
	34-36	3.47	3.13	3.84
	37-39	3.65	3.29	4.05
	40-42	3.35	3.00	3.73
	43-44	3.08	2.75	3.44
	45+	2.50	2.23	2.80
Previous opposite sex marriage				
	No	1		
	Yes	0.42	0.39	0.46

Table A2: Event history model on the risk of first same-sex marriage formation, men and women in Sweden, including the interaction of calendar period and sex (risks relative to that of men in 2002), and control for educational attainment and study enrollment

		Relative risks	95% C. I.	
Period - Same sex men				
	1995	2.39	1.90	3.01
	1996	0.96	0.73	1.26
	1997	0.87	0.66	1.14
	1998	0.77	0.58	1.02
	1999	0.86	0.65	1.14
	2000	1.06	0.82	1.37
	2001	0.91	0.69	1.19
	2002	1		
	2003	1.07	0.83	1.38
	2004	1.32	1.04	1.69
	2005	1.04	0.80	1.35
	2006	1.20	0.94	1.54
	2007	1.06	0.82	1.36
	2008	1.14	0.88	1.46
	2009	1.28	1.00	1.63
	2010	1.67	1.33	2.11
	2011	1.55	1.23	1.96
	2012	1.40	1.10	1.77
Period - Same sex women				
	1995	1.61	1.23	2.11
	1996	1.11	0.83	1.49
	1997	1.16	0.87	1.54
	1998	0.97	0.72	1.31
	1999	1.23	0.93	1.63
	2000	1.41	1.08	1.85
	2001	1.89	1.47	2.42
	2002	1.74	1.35	2.24
	2003	2.43	1.92	3.08
	2004	2.59	2.06	3.27
	2005	3.06	2.44	3.83
	2006	3.49	2.80	4.35
	2007	3.41	2.73	4.25
	2008	4.51	3.64	5.57
	2009	4.86	3.94	6.00
	2010	5.47	4.44	6.73
	2011	6.24	5.08	7.66
	2012	5.59	4.54	6.87
Parity				
	0	1		
	1	0.14	0.12	0.15

	2	0.04	0.04	0.05
	3+	0.04	0.03	0.05
Age				
	16-18	0.02	0.01	0.03
	19-21	0.13	0.10	0.15
	22-24	0.47	0.41	0.54
	25-27	1		
	28-30	1.80	1.63	1.99
	31-33	2.75	2.49	3.04
	34-36	3.39	3.06	3.76
	37-39	3.62	3.26	4.03
	40-42	3.36	3.01	3.76
	43-44	3.11	2.78	3.49
	45+	2.54	2.26	2.85
Previous opposite sex marriage				
	No	1		
	Yes	0.41	0.37	0.44
Education				
	Primary	1.04	0.95	1.14
	Secondary	1		
	Tertiary	1.46	1.39	1.55
	Enrolled	1.14	1.04	1.26
	Missing	0.35	0.24	0.53

Table A3: Event history model on divorce risks in first marriages, men and women in same-sex marriages and women in opposite-sex marriages, with interaction of period and type of marriage (risks relative to that of men in same-sex marriages in 2002)

	Relative risks	95% C. I.	
Period - Same sex men			
1995	0.00	0	.
1996	0.00	0	.
1997	0.00	0	.
1998	2.08	1.14	3.78
1999	1.17	0.61	2.23
2000	0.95	0.50	1.81
2001	0.98	0.53	1.81
2002	1		
2003	1.33	0.77	2.27
2004	0.92	0.52	1.61
2005	0.95	0.55	1.65
2006	1.52	0.93	2.50
2007	0.69	0.39	1.21
2008	0.76	0.44	1.31
2009	0.73	0.42	1.25
2010	0.62	0.36	1.08
2011	0.73	0.43	1.24
2012	0.54	0.31	0.95
Period - Same sex women			
1995	0.00	0	.
1996	0.00	0	.
1997	0.00	0	.
1998	1.56	0.72	3.38
1999	1.99	1.06	3.74
2000	2.88	1.69	4.93
2001	2.53	1.50	4.27
2002	1.42	0.81	2.48
2003	0.96	0.54	1.72
2004	1.08	0.63	1.85
2005	1.22	0.73	2.04
2006	1.23	0.75	2.02
2007	1.17	0.72	1.91
2008	0.97	0.60	1.59
2009	1.00	0.62	1.62
2010	1.24	0.78	1.97
2011	1.37	0.87	2.16
2012	1.11	0.70	1.75
Period - Opposite sex women			
1995	0.19	0.10	0.34
1996	0.44	0.28	0.68

	1997	0.63	0.41	0.97
	1998	0.63	0.41	0.96
	1999	0.69	0.45	1.05
	2000	0.74	0.49	1.13
	2001	0.74	0.49	1.13
	2002	0.73	0.48	1.12
	2003	0.66	0.44	1.01
	2004	0.64	0.42	0.97
	2005	0.63	0.41	0.95
	2006	0.64	0.42	0.98
	2007	0.64	0.42	0.97
	2008	0.66	0.43	1.00
	2009	0.67	0.44	1.02
	2010	0.71	0.47	1.08
	2011	0.69	0.45	1.04
	2012	0.67	0.44	1.02
Parity				
	0	1		
	1	0.47	0.45	0.48
	2	0.44	0.43	0.45
	3+	0.47	0.46	0.49
Age				
	16-18	3.54	2.59	4.83
	19-21	2.72	2.55	2.91
	22-24	1.66	1.60	1.73
	25-27	1		
	28-30	0.65	0.63	0.67
	31-33	0.47	0.46	0.49
	34-36	0.39	0.37	0.40
	37-39	0.33	0.32	0.35
	40-42	0.29	0.28	0.30
	43-44	0.26	0.25	0.27
	45+	0.22	0.20	0.23
Duration of marriage				
	0-1 year	0.14	0.13	0.15
	1-2 years	0.35	0.33	0.36
	2-3 years	0.76	0.74	0.78
	3-4 years	1		
	4-6 years	1.23	1.20	1.26
	7-10 years	1.47	1.42	1.51
	11+ years	1.65	1.59	1.72
Premarital childbearing				
	No	1		
	Yes	1.17	1.15	1.19

Table A4: Event history model on divorce risks in first marriages, men and women in same-sex marriages and women in opposite-sex marriages, with interaction of period and type of marriage (risks relative to that of men in same-sex marriages in 2002), and control for educational attainment and study enrollment

	Relative risks	95% C. I.	
Period - Same sex men			
1995	0.00	0	.
1996	0.00	0	.
1997	0.00	0	.
1998	2.02	1.11	3.67
1999	1.15	0.61	2.20
2000	0.96	0.50	1.82
2001	0.98	0.53	1.81
2002	1		
2003	1.32	0.77	2.26
2004	0.92	0.52	1.63
2005	0.96	0.55	1.66
2006	1.54	0.94	2.54
2007	0.69	0.39	1.22
2008	0.77	0.44	1.32
2009	0.74	0.43	1.27
2010	0.64	0.37	1.10
2011	0.76	0.45	1.28
2012	0.57	0.33	0.99
Period - Same sex women			
1995	0.00	0	.
1996	0.00	0	.
1997	0.00	0	.
1998	1.51	0.70	3.28
1999	1.94	1.03	3.66
2000	2.88	1.68	4.92
2001	2.56	1.52	4.31
2002	1.45	0.83	2.53
2003	0.99	0.55	1.76
2004	1.13	0.66	1.94
2005	1.29	0.78	2.15
2006	1.32	0.80	2.16
2007	1.22	0.75	1.99
2008	1.03	0.63	1.67
2009	1.07	0.66	1.73
2010	1.33	0.84	2.11
2011	1.47	0.94	2.32
2012	1.21	0.76	1.90
Period - Opposite sex women			
1995	0.21	0.12	0.37

	1996	0.48	0.31	0.75
	1997	0.69	0.45	1.05
	1998	0.69	0.45	1.05
	1999	0.75	0.49	1.14
	2000	0.82	0.54	1.24
	2001	0.81	0.53	1.23
	2002	0.81	0.53	1.23
	2003	0.73	0.48	1.12
	2004	0.73	0.48	1.11
	2005	0.72	0.47	1.09
	2006	0.74	0.49	1.12
	2007	0.72	0.47	1.09
	2008	0.74	0.49	1.13
	2009	0.77	0.50	1.17
	2010	0.81	0.53	1.23
	2011	0.79	0.52	1.20
	2012	0.78	0.51	1.19
Parity				
	0	1		
	1	0.49	0.47	0.50
	2	0.47	0.46	0.48
	3+	0.49	0.48	0.51
Age				
	16-18	2.47	1.80	3.37
	19-21	2.13	1.99	2.28
	22-24	1.48	1.42	1.54
	25-27	1		
	28-30	0.71	0.68	0.73
	31-33	0.53	0.52	0.55
	34-36	0.45	0.44	0.47
	37-39	0.40	0.38	0.41
	40-42	0.35	0.33	0.36
	43-44	0.31	0.29	0.32
	45+	0.25	0.24	0.27
Duration of marriage				
	0-1 year	0.15	0.14	0.16
	1-2 years	0.36	0.35	0.37
	2-3 years	0.77	0.75	0.80
	3-4 years	1		
	4-6 years	1.20	1.17	1.24
	7-10 years	1.40	1.36	1.45
	11+ years	1.56	1.50	1.62
Premarital childbearing				
	No	1		
	Yes	1.04	1.03	1.06
Education				

Primary	1.74	1.69	1.79
Secondary	1		
Tertiary	0.70	0.69	0.72
Enrolled	1.15	1.12	1.19
Missing	1.21	1.05	1.39

Table A5: Re-categorized models with broader calendar-period categories in same-sex marriage formation. All models use the same regression specifications as in Appendix Table A1 and only differ by the aggregation of calendar year and choice of reference categories. P-values refer to pairwise tests of differences between calendar-year group and type of marriage.

Reference category (right)	Model 1		Model 2		Model 3		Model 4		Model 5		Model 6	
Covariate within model (below)	Male same-sex, 1995-2002		Male same-sex, 2003-2008		Male same-sex, 2009-2012		Female same-sex, 1995-2002		Female same-sex, 2003-2008		Female same-sex, 2009-2012	
	RR	p-value	RR	p-value	RR	p-value	RR	p-value	RR	p-value	RR	p-value
Male same-sex, 1995-2002	1		0.894	0.013	0.733	0.000	0.853	0.001	0.376	0.000	0.247	0.000
Male same-sex, 2003-2008	1.119	0.013	1		0.820	0.000	0.954	0.324	0.420	0.000	0.276	0.000
Male same-sex, 2009-2012	1.364	0.000	1.219	0.000	1		1.164	0.002	0.513	0.000	0.337	0.000
Female same-sex, 1995-2002	1.172	0.001	1.048	0.324	0.859	0.002	1		0.441	0.000	0.290	0.000
Female same-sex, 2003-2008	2.661	0.000	2.378	0.000	1.951	0.000	2.270	0.000	1		0.657	0.000
Female same-sex, 2009-2012	4.048	0.000	3.618	0.000	2.968	0.000	3.453	0.000	1.521	0.000	1	

Notes: We observe monotonic increases in marriage formation across all calendar periods for both men and women. All changes in male marriage risks over time, and for female marriages risks over time, are statistically significant at the five-percent level. Female marriage risks are statistically significantly higher than male risks for all periods.

Appendix Table A6: Re-categorized models with broader calendar-period categories in divorce patterns. All models use the same regression specifications as in Appendix Table A3 and only differ by the aggregation of calendar year and choice of reference categories. P-values refer to pairwise tests of differences in divorce risks between calendar-year group and type of marriage.

	Model 1		Model 2		Model 3		Model 3		Model 4		Model 5		Model 6		Model 7		Model 8	
Reference category (right)	Male same-sex 1995-2002		Male same-sex 2003-2008		Male same-sex 2009-2012		Female same-sex 1995-2002		Female same-sex 2003-2008		Female same-sex 2009-2012		Female opposite-sex 1995-2002		Female opposite-sex 2003-2008		Female opposite-sex 2009-2012	
Covariate within model (below)	RR	p-value	RR	p-value	RR	p-value	RR	p-value	RR	p-value	RR	p-value	RR	p-value	RR	p-value	RR	p-value
Male same-sex, 1995-2002	1		0.925	0.539	1.431	0.011	0.598	0.000	1.010	0.935	0.948	0.640	1.236	0.045	1.345	0.005	1.257	0.029
Male same-sex, 2003-2008	1.081	0.539	1		1.548	0.000	0.646	0.000	1.092	0.345	1.025	0.779	1.336	0.000	1.455	0.000	1.360	0.000
Male same-sex, 2009-2012	0.699	0.011	0.646	0.000	1		0.418	0.000	0.706	0.002	0.662	0.000	0.863	0.123	0.940	0.513	0.879	0.172
Female same-sex, 1995-2002	1.674	0.000	1.548	0.000	2.395	0.000	1		1.690	0.000	1.586	0.000	2.068	0.000	2.251	0.000	2.104	0.000
Female same-sex, 2003-2008	0.990	0.935	0.916	0.345	1.417	0.002	0.592	0.000	1		0.938	0.401	1.224	0.001	1.332	0.000	1.245	0.000
Female same-sex, 2009-2012	1.055	0.640	0.976	0.779	1.510	0.000	0.631	0.000	1.066	0.401	1		1.304	0.000	1.420	0.000	1.327	0.000
Female opposite-sex, 1995-2002	0.809	0.045	0.748	0.000	1.158	0.123	0.484	0.000	0.817	0.001	0.767	0.000	1		1.089	0.000	1.018	0.153
Female opposite-sex, 2003-2008	0.743	0.005	0.687	0.000	1.064	0.513	0.444	0.000	0.751	0.000	0.704	0.000	0.919	0.000	1		0.935	0.000
Female opposite-sex, 2009-2012	0.795	0.029	0.735	0.000	1.138	0.172	0.475	0.000	0.803	0.000	0.754	0.000	0.983	0.153	1.070	0.000	1	

Notes on statistical differences:

Female same-sex unions have significantly higher divorce risk than opposite sex unions in all calendar periods.

Male same-sex unions have higher divorce risks than opposite sex unions in 1995-2008, but not in 2009-2012.

Male same-sex unions have divorce risks in 2009-2012 that are statistically lower than those for men in 1995-2008.

Female same-sex unions have a decrease in divorce risks from 1995-2002 to 2003-2012 that is statistically significant.

Male same-sex unions have lower divorce risks than female same-sex unions in 1995-2002 and 2009-2012, but not in 2003-2008.

Figure A1: Relative risks of first marriage formation in Sweden, by age group, 1995-2012. Separate models for same-sex and opposite-sex marriage formation. Risks are given relative to that of the age group with the highest marriage intensity. Risks are standardized for calendar year, parity, and the experience of any previous opposite-sex marriage (for same-sex marriages).

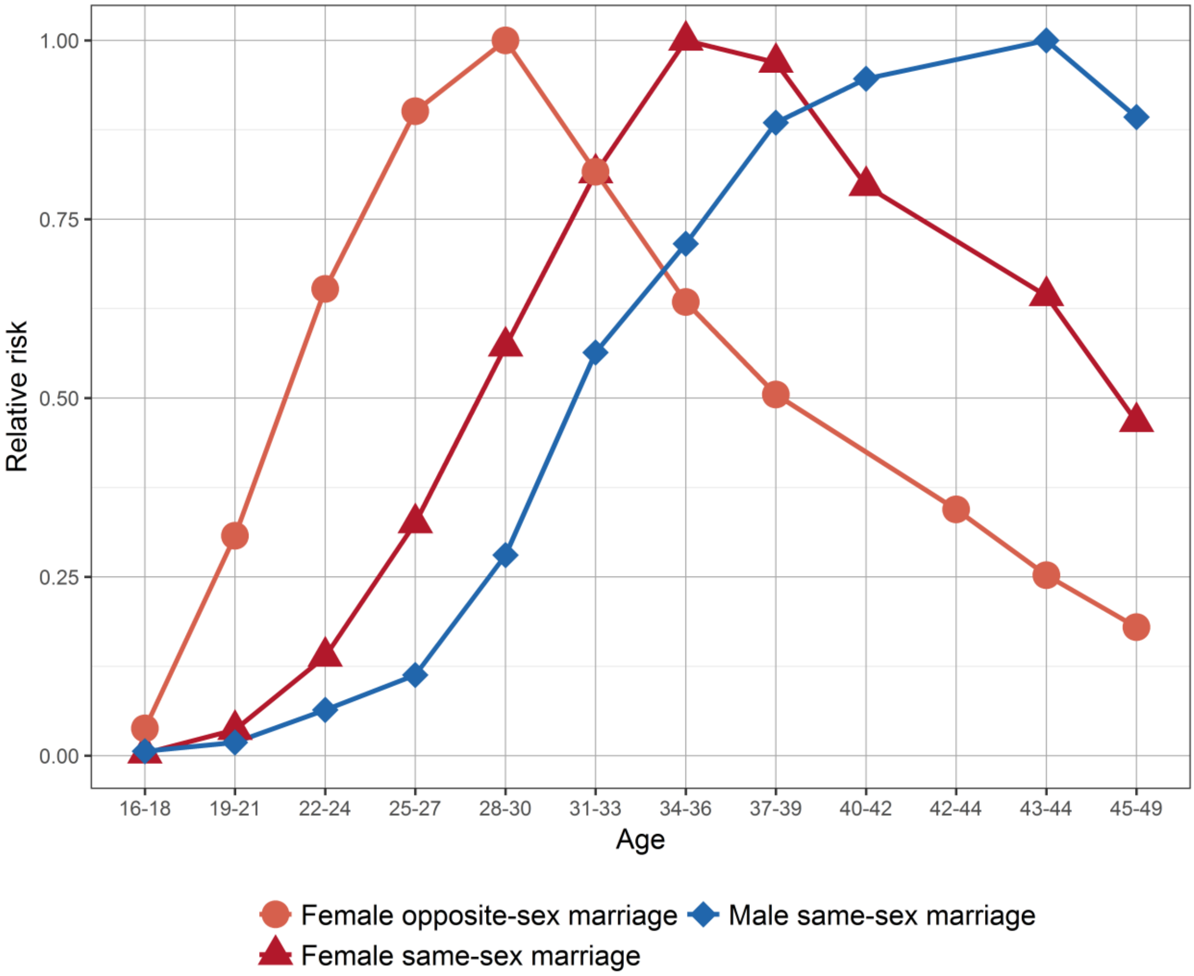

Source: Swedish register data, authors' own calculations

Figure A2: Relative risks of first opposite-sex marriage formation for women in Sweden, by calendar year and parity, 1995-2012. Risks relative to that of childless women in 2002. Risks are standardized for age.

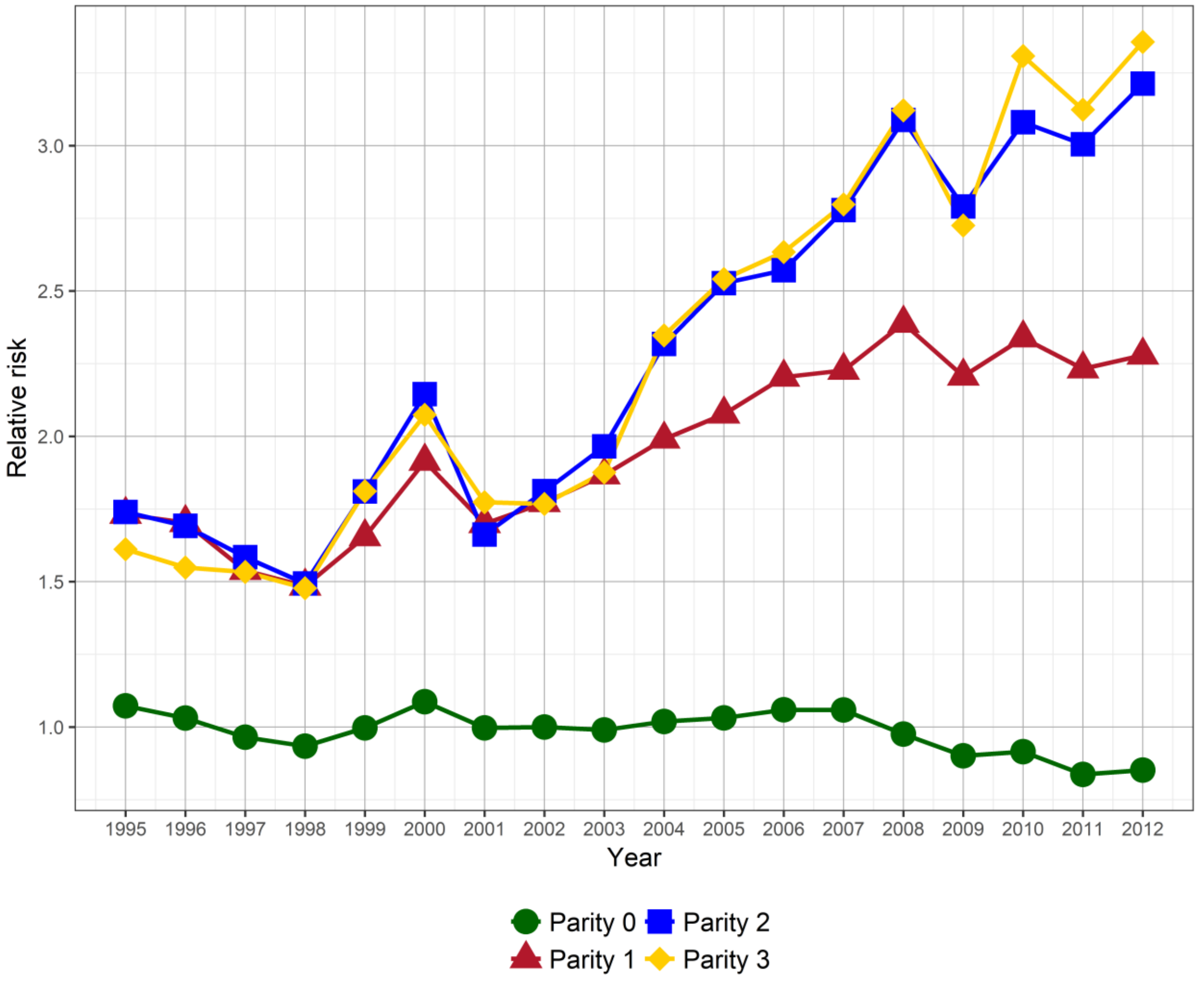

Source: Swedish register data, authors' own calculations

Figure A3: Proportion of same-sex marriages of women entered between 1995 and 2008, by childbearing experience within five years from marriage formation.

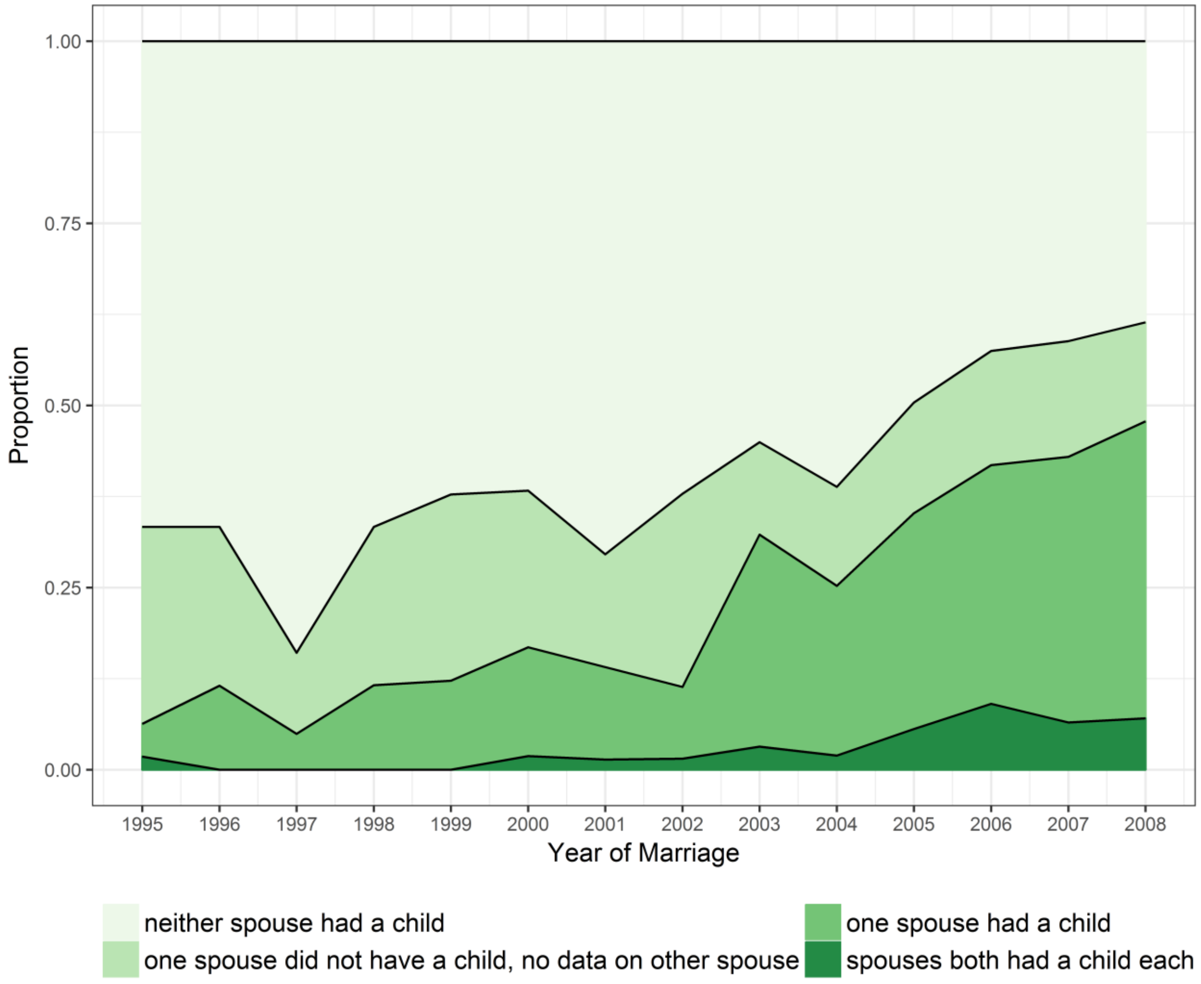

Source: Swedish register data, authors' own calculations.

Note: In order for a woman to be included as an index person she has to be Swedish-born and younger than 49. Women in Figure A3 with missing data on her partner include those whose partner did not meet these criteria. The corresponding proportion of men where either or both spouses had a child is below 5% for marriages formed in 2006-2008.
